# New Zealand and the Supervision of Its Meat Supplies

**Published:** 1906-10-13

**Authors:** 


### The Food of the Masses.

### NEW ZEALAND AND THE SUPERVISION OF ITS MEAT SUPPLIES.

### "The Price of Concealment."

A large and important industry has been nearly ruined by a reckless indulgence in a policy of concealment. For although it is not necessary to lend a believing ear to all the statements which have been made on the subject of the American Meat Supply, the strenuous efforts made to prevent efficient inspection of the products and premises form the strongest indictment against it. If the conditions are ideal there can be no objection to efficient inspection either by a Government nominee, by a nominee of a public body, or for that part by any one expressing a desire. On the other hand, a novelist and story-teller is scarcely the proper person to give unbiassed views of a business of this kind.

The public are entitled to know what they are eating and to be protected against food poisoners. They are entitled to receive adequate value for the money they expend on food—to be assured that when they ask for food they shall not receive masses of putrefaction so artfully prepared as to defy detection by any method except the deplorable physiological results which follow the consumption of such articles. How marked these were in the South African campaign, no one can ever exactly tell, but the evidence was such that many tons of canned garbage supplied for feeding soldiers were destroyed after report from duly qualified officers. In many instances it was the product of home manufacture, and not of American importations. A policy of concealment inevitably reacts and carries ruin and dismay among the dealers and disease among the public. These food products form the basis of a great national industry upon which vast numbers of the population rely for sustenance and, if properly handled, would be to the benefit of mankind. Our peoples' food largely depends on the supply from abroad, and foreign and colonial products cannot be delivered in this country by the Marconi system. It is estimated that last year the importation of beef, mutton, bacon, hams, pork, rabbits and fish in bulk amounted to 314,575 tons, and the tinned food entering this country was 31,085 tons. To this has to be added 120,000 tons as the amount of dairy produce imported and a quantity of eggs that could not be numbered for multitude. This foreign food supply is a necessity and, therefore, it is all the more urgent that a firm line should be drawn between the garbage prepared under unhygienic factory conditions closed to inspection, and those products which, on the other hand, are produced in works open to inspection, in healthy and specially built premises, and, as it were, under the public eye.

### The Method in New Zealand.

To this latter class belong the products of New Zealand which for many years past has done its utmost to ensure the perfect purity of its meat output. The trade with New Zealand is a large one. In 1903 nearly 5,000,000 carcasses of mutton and lamb were exported; in 1904 and in 1905 there was some falling off in exportation, partly because more new country was being opened up and the sheep were being used to stock the new farms. This year, however, exportation is resuming its normal standard of over 5,000,000 carcasses, mostly of frozen mutton. In addition to mutton considerable quantities of frozen beef are exported. The point to be emphasised about New Zealand is the thorough Government inspection of all works devoted to this industry. To each one of the meat export works is attached a qualified veterinary surgeon trained in an English or Scotch college, with expert assistants qualified by passing a local examination. This official has to carry out the regulations of the Government laid down in the Slaughtering and Inspection Act of 1900. The legislation enacted in New Zealand is not "scare" legislation, but has been adopted by the colony purely in the best interest of the meat trade upon which the prosperity of the country largely depends. The public cannot fail to take note of this enlightened legislation, and as a consequence the colony will benefit. The chief sections of the Act provide that every part of such works shall be open to the Government inspector, who may at any time enter into "slaughtering places, ship or conveyance, and there do whatever he deems necessary to prevent any such place, ship, or conveyance which, in his opinion, is in any way insanitary, defective, or unsuitable, from being used for the collecting, slaughtering, or carriage of stock or meat." Section 34 provides that "it shall not be lawful for any person to slaughter any stock in the abattoir or meat export slaughtering-house without the written authority of an inspector." The carcasses must be inspected before any meat is removed either for human consumption or for export until the inspector declares it to be free from disease. These regulations are strictly enforced.

### No Bribery and Corruption.

The Government servants in New Zealand are not controlled by powerful corporations and trusts, which have proved so disastrous to efficient inspection in the United States. In New Zealand there is no combine. The companies are keen competitors and any case of corruption or bribery of officials would at once be detected and visited with severe consequences. To still further prevent this the inspectors are moved from one factory to another much in the same way as are our own revenue officials and coastguardsmen. Each carcass, along with its internal organs, is examined for disease immediately after slaughter. It is from these that the best evidence of fitness or unfitness is obtained. Only the absolutely pure and healthy carcases are permitted to leave the establishments for consumption, and any carcase found to be diseased, or even suspected of being unhealthy and unfit for human food, is immediately destroyed. For the loss of condemned carcasses owners are adequately compensated by the Government. This liberal treatment entirely removes any temptation to evade the law as regards the purity of the meat.

### Inspection on Shipment.

New Zealand meat is again inspected on shipment and not allowed to be stored without a certificate stating that it is in good condition. Regulations of this nature in our own country frequently become, by reason of routine officialism, mere dead letters, but in New Zealand this, it is insisted upon, does not happen. The inspection, so far from being performed in a perfunctory and routine manner, is a very live business. So thoroughly is this method of examination carried out at the port of export that one would imagine little was left to be done by the authorities at home except to ascertain how the carcasses had been able to stand the long voyage.

### Freezing the Carcass.

As soon as the animal is killed and the natural heat has left its body it is immediately frozen, and remains in that stationary condition until it arrives in England. Frozen meat if kept in cold store for too long a period has not the lustrous appearance of freshly killed meat; but if properly treated it ought to retain its bloom—a point upon which buyers insist. The difficulty of proper inspection at home ports forms an objection to frozen meat, and points to the absolute necessity of having each of the carcasses from whichever country they may be imported supervised at the port of export exactly in the same detailed manner as is done by the authorities of New Zealand. Cold or freezing will not render a carcass sterile, for certain germs of putrefaction resist low temperatures, a resistance which does not in any way prevent the preservative action of cold. The low temperature arrests the development of decay, and the flesh remains fresh until it is exposed for sale, or when it gradually thaws. All frozen meat should be cooked on the Warren system. This involves putting each joint into cold water in a stewpan and leaving it there until the water begins to boil. It should then be taken out and roasted for half to three-quarters of an hour. Frozen meat, whether beef or mutton, treated in this way cannot be distinguished from fresh meat. All the juices are retained, too, and the meat for food purposes is improved by the process.

### How to Cook Frozen Mutton.

People unfortunately generally use New Zealand mutton too soon after thawing. After being taken out of the cold air and exposed to the ordinary atmosphere and thawed, the meat is not ready for cooking until, like ordinary English meat, it has hung from three to five days, according to the season and temperature, like English meat. Then it becomes tender and suitable for food. If this be done there is no reason why New Zealand mutton should not be equal in point of tenderness and quality to English mutton. It should be kept in a dry, cool (not cold), larder, and it is of great importance that joints should be so hung that the juices do not drip from the cut end. It is advisable to point out that although New Zealand meat when hung loses its bright appearance more rapidly than meat slaughtered in this country, this in no way affects its freshness or quality.

Frozen meat, when thawed and exposed on a hot day, may become tainted, as English meat does. It is frequently sold at a cheap rate in shops in the poorer neighbourhoods, and thus provides a very important article of food for the masses, who can purchase it at a much cheaper rate than is possible with English meat. When it is properly treated and kept under suitable conditions it is an agreeable, nutritious, and wholesome food. This leads to the noxious practice of substitution. Very large quantities of New Zealand meat are sold by the high-class trade, much of it as "English." There is no reason why, when the carcase has become unsound from over-exposure, bad treatment, or long delay in the hot, close, and dirty shops which abound in the poorer neighbourhoods of London, the blame should be laid at the door of the imported article; concerning the wholesomeness of which it may be said to have undergone all the precautions that human ingenuity and science can invent to make it suitable for human consumption. In "market streets" decayed pieces of foreign foodstuffs are said to be rejuvenated with a paint brush and sold "for what they will fetch." It is common in the summer to see a butcher offering a joint at 2d. a pound, and sometimes even lower, for the purpose of clearing out, often giving the poor people a good dinner when otherwise they could not obtain it. In this respect the Londoner is much better off than the country people, who can never get their meat under 6d. a pound.

### New Zealand's Tinning Industry.

We have devoted a considerable amount of space in considering certain brands of tinned food-stuffs manufactured in England, and on the whole they have stood well the tests we have applied to them. It is in this class of meat products that inspection on the spot is more than ever necessary, for when once "tinned" the quality of the goods is beyond inspection, and in practice must be judged of by examining the outside of the tin. If the tins are seen to be blown or bulged it is assumed that the contents have become putrefied. The garbage manufacturer is not at a loss. He pricks the tins, lets the gas escape, boils up the contents, resolders, and these "do-overs" are ready for the market once more. No test can be applied to discover these iniquities except the poisoning which ensues after they are taken into the body. The examination of tins at home is absolutely useless as a preventive measure, and here again the arrangements for tinned meats manufactured in New Zealand may serve as a model for other countries engaged in the same trade to adopt.

### Inspection on the Spot.

No matter what the inspection at home is, the consumers of tinned meats must realise that they are mainly dependent on the officials on the spot. At the Grocers' Exhibition it was found that tinned fodo products were a drug on the market, so little has been the demand for this article of consumption. Dealers are fully stocked with American produce of which the public has heard so much that they do not greatly favour it. They were, however, fully interested in our colonial products coming from countries where inspection is under Government régime, where the public servants are fully trained and qualified by experience and practice, and are beyond the reach of bribery and corruption, influences which have done so much to discredit and well nigh ruin an important national industry. The buyers at the Grocers' Exhibition were interested in products signed and sealed by Government stamp, every item of which had been carefully examined and verified. They have been taught a severe lesson and obviously manifested a lively interest in New Zealand products. Good seed was sown by the New Zealand authorities at the Grocers' Exhibition with their kindly and courteous manner, entirely destitute of the style of the pushful salesman so common a nuisance in such trade exhibitions.

### Export Certificate.

All meat leaving New Zealand must be branded with a tag or label—the certificate of the veterinary surgeon. This meat (see Section 41 of the Act already alluded to) "shall not be deemed to be duly branded unless each tin and also each case or other package in which the goods are packed are distinctly branded with the following particulars:—(1) The words "New Zealand"; (2) name of the owner or exporter and his registered trade-mark; (3) the true name and description of the contents. In Section 42 of the Act "every slaughtering-place, sale-yard, conveyance or other place where stock are confined, or being carried, shall at all times, to the satisfaction of the inspector, be kept efficiently lighted, ventilated, cleansed, drained, and provided with a sufficient water supply, and no offal, filth, or refuse shall be allowed to remain thereon for more than 24 hours." A regulation of this kind, properly carried out, fulfils most that is required in the way of cleanliness, and if it had been applied to the Chicago abattoirs no such scandal and scare as has moved the world would have become possible.

### No Overcrowding in New Zealand Works.

There is still another point. Chicago is known as the "packing town." Into it is concentrated the enormous industry of a whole continent within a comparatively small area, overcrowding such as is impossible in New Zealand being a normal condition. In New Zealand the freezing works are situated in the open country, having plenty of air and other healthy surroundings. The employés do not come from the filthy dens of polluted cities, but are well housed, healthy and well fed. The butchers are a fine body of men, earning good wages—a slaughter-man will earn about £3 per week. These points indicate the practical application of sanitary knowledge, and naturally react on the wholesomeness of the food which is being prepared for public consumption in this country. New Zealand is a land of running waters with an insular climate—the climate of the South of England, but with more sunshine and a steady rainfall. The cattle upon a thousand hills are plenteously pastured, and killed when in the pink of condition.

### The Energy of the High Commissioner and His Staff.

The export industry has been greatly helped by the energy and loyalty of the High Commissioner, Mr. Reeves. At the time of the Chicago scare, when the trading prosperity of New Zealand was partially threatened, the High Commissioner considered that it would be a good opportunity to bring New Zealand goods before the notice of the British public. He, therefore, took steps and invited New Zealand packers to send home goods for show at the Grocers' Exhibition in the Agricultural Hall, Islington. Seven of the meat-packing companies responded to this call, and their display made a very imposing addition to this 14th Annual International Exhibition. The meat business of New Zealand is mostly a frozen meat industry, tinning and canning being a side industry. The exports amount, as we have mentioned, to some 4,000,000 carcases of mutton and lamb annually, nearly all of which finds purchasers and consumers in this country.

### Exhibitors at the Grocers' Exhibition.

The companies that exhibited in response to the High Commissioner's invitation were: The Gear Meat Preserving Co., Ltd., Wellington; the Wanganui Meat Freezing Co., Ltd., Wanganui; the Christchurch Meat Co., Ltd., Christchurch; the South New Zealand Meat Preserving Co., Winton; the Wellington Meat Export Co., Ltd., Wellington; W. E. Tait, Woodlands Preserving Works, Invercargill; and Irvine and Stevenson, St. George Preserving Co., Ltd., Dunedin. They deserve credit for their enterprise and for the success which attended their efforts. Of the samples taken at random from these exhibitors' goods we examined "ox-tail soup" from the Gear Meat Preserving Co., Ltd., and found it of excellent quality. The same may be said of the "jugged hare" of Irvine and Stevenson, St. George Preserving Co. The sheeps' tongues from the Christchurch Meat Preserving Co. (Aorangi Brand), and those from Wanganui Meat Freezing Co., were in every way satisfactory and delicious. They were soft and tender, and maintained their natural flavour as if they had just been cooked—qualities which it is very rare to find.

